# Enzymatic Kinetic Resolution of 2-Piperidineethanol for the Enantioselective Targeted and Diversity Oriented Synthesis †

**DOI:** 10.3390/ijms17010017

**Published:** 2015-12-24

**Authors:** Dario Perdicchia, Michael S. Christodoulou, Gaia Fumagalli, Francesco Calogero, Cristina Marucci, Daniele Passarella

**Affiliations:** Dipartimento di Chimica, Universita degli Studi di Milano, Via Golgi 19, 20133 Milano, Italy; michalis.christ@gmail.com (M.S.C.); gaia.fumagalli@unimi.it (G.F.); fcalogero87@gmail.com (F.C.); cristina.marucci@unimi.it (C.M.)

**Keywords:** 2-piperidineethanol, piperidine alkaloids, enzymatic resolution, alkaloid synthesis

## Abstract

2-Piperidineethanol (**1**) and its corresponding *N*-protected aldehyde (**2**) were used for the synthesis of several natural and synthetic compounds. The existence of a stereocenter at position 2 of the piperidine skeleton and the presence of an easily-functionalized group, such as the alcohol, set **1** as a valuable starting material for enantioselective synthesis. Herein, are presented both synthetic and enzymatic methods for the resolution of the racemic **1**, as well as an overview of synthesized natural products starting from the enantiopure **1**.

## 1. Introduction

**Figure 1:**
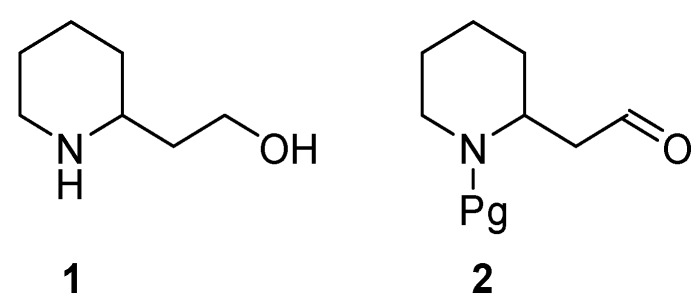
Structure of 2-piperidineethanol (**1**) and the corresponding *N*-protected aldehyde (**2**).

2-Piperidineethanol (**1**) (Figure 1) is a chiral piperidine derivative bearing two functional group suitable for diversified elaborations of the molecule.

With more than 10,000 molecules listed in SciFinder, contain the substructure of 2-piperidineethanol keeping the stereocentre in position 2 of piperidine, its potentiality is self-evident as a valuable chiral starting material in organic synthesis. In racemic form, it is a quite cheap compound since it is synthesized, on an industrial scale, by catalytic hydrogenation of 2-(2-hydroxyethyl)pyridine [1]; on the contrary, the enantiopure form is expensive and less available.

*N*-protected aldehyde (**2**), easily obtained by controlled oxidation of the parent alcohol, is the derivative of 2-piperidineethanol usually used to a great extent in synthesis.

The racemic compound is clearly related to the main exploitations but in the last decades there was a growing utilization of the enantiopure form, mostly concerning the synthesis of new leads in medicinal chemistry and the total synthesis of natural compounds.

*Per se*, it is utilized in the industrial plants for CO_2_ capture [2]; as racemic starting compound was used in the synthesis of the catalysts for Ziegler–Natta polymerization [3]. It is used as scaffold for the synthesis of insect and mite repellents [4] as Icaridin (trade name Saltidin) [5]; *tert*-butyl carbamate (*N*-Boc) protected 2-piperidineethanol was patented as anthelminthic agents for humans and animals [6]. In medicinal chemistry, several leads were synthesized from racemic **1**; it is worth citing the synthesis of a new orexin receptor antagonist [7], of a new class of non-opiate antinociceptive agents [8], of (aryloxyalkyl)piperidines as calcium channel antagonists [9] and of sulfonamide derivatives as 5-HT_7_ receptor (5-hydroxytryptamine receptor 7) antagonists [10]. In some cases, a further improvement of activity was granted with the proper enantiomer, for example, the synthesis of aminoindane compounds as sodium channel inhibitors and TRPV1 receptor (transient receptor potential cation channel subfamily V member 1) activator for the treatment of pain [11], of a novel non-xanthine adenosine A1 receptor antagonist [12], of dermal anesthetic agents [13], of gonadotropin-releasing hormone antagonists [14], of a new inhibitor of cyclin-dependent kinases [15], of non-peptide quinolone GnRH (gonadotropin-releasing hormone) receptor antagonists [16] and of selective thrombin inhibitors [17].

Nature is the prime developer of compounds with a piperidine backbone with many structurally-interesting alkaloids; the wide range of biological activities in this family of natural products make them ideal targets for total synthesis. A selection of synthesis of alkaloids starting from enantiopure **1** is displayed at the end of this review.

## 2. Enantiopure 2-Piperidineethanol: Resolution *versus* Synthesis

Some research groups took into account the enantioselective synthesis of **1** or aldehyde **2**; an early strategy was established on intermolecular nitrone cycloadditions to chiral allyl ethers [18,19] obtaining benzyl carbamate (*N*-Cbz) **2**. Asymmetric allylation was the next synthetic tool using a chiral auxiliary [20,21]; a synthetic sequence involving a Maruoka asymmetric allylation with a chiral catalyst [22] was successful in obtaining *N*-Boc **2**. Recently, a straightforward organocatalyzed intramolecular aza-Michael reaction accomplished the synthesis of either *N*-Boc or *N*-Cbz **2** in high enantiomeric excess (e.e.) [23,24]. To obviate to the numerous synthetic steps necessary in the synthesis of **1** from scratch was also taken advantage of the homologation of the commercially-available *N*-(Boc)-*S*-pipecolic acid by carboxyl reduction, modified Swern oxidation, Wittig olefination, and enol ether acidic hydrolysis [25].

Resolution of the racemic mixture certainly provides practical benefits, considering the low price and good availability of **1**, although suffers from the disadvantage that only half of the starting material is transformed into the desired product. Resolution can then offer the most cost efficient route to the enantiomerically pure chiral synthon. Indeed, it was the first method used to obtain the enantiopure isomer by careful recrystallization of the diasteroisomeric d-10-camphorsulfonate salt from ethanol-ether [26]. This method generally provided the alcohol in low yield, since there was a strong tendency for the racemic salt to separate, rather than the diastereoisomeric forms and numerous sequential recrystallizations were required to achieve enantiomeric excess of 95%. The procedure was subjected at slight modifications as it was repeated by several research groups and, although with some drawbacks, was used for the production of 12 kg of (*R*)-(−)-2-piperidineethanol [12]. Recently, the pushing necessity for a better resolving agent, brought about the development of two patents that utilized either *N*-sulphonyl pyroglutamic acid [27] or *N*-acetyl-l-leucine [28]: a single crystallization of the corresponding diastereoisomeric salt with **1**, was enough to achieve high enantiomeric excess (86% and 95%, respectively) and higher with the second crystallization (99% and 98%, respectively).

### Enantiopure 2-Piperidineethanol by Enzymatic Kinetic Resolutions

**Scheme 1:**
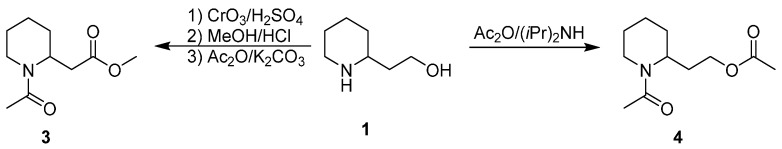
Synthesis of 2-piperidineethanol derivatives suitable for hydrolysis by pig liver esterase. *i*Pr: isopropyl.

**Scheme 2:**
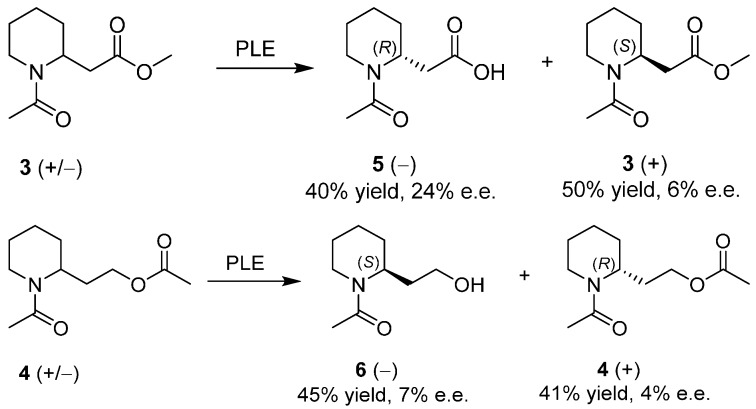
Enzymatic kinetic resolutions of racemic **3** and **4**. PLE: pig liver esterase; e.e.: enantiomeric excess.

Enzymatic kinetic resolutions of racemates have been widely exploited in the past years for the preparation of enantiopure compounds [29,30,31]. Despite the breakthroughs achieved through the ability of enzymes to selectively react with only one of the enantiomers from a racemic mixture, there are only few examples reported for the resolution of **1**, each one related to the reactivity at the hydroxyl group. It was a difficult task, due to the fact that the molecule is a conformationally flexible and primary alcohol located two carbons away from the molecule stereocenter. An early reference approached the resolution of several racemic piperidine derivatives [32] with either a stereocenter in position 2 or 3, exploiting the hydrolysis of an ester group by pig liver esterase (EC 3.1.1.1). With **1** two strategies were tested (Scheme 1) by the synthesis of esters **3** and **4**:

They were subjected to pig liver esterase hydrolysis at pH 7.0 to avoid the possibility of competing chemical hydrolysis or epimerization under the higher pH conditions; reactions were stopped after 50% conversion: results are reassumed in Scheme 2 showing a generally low enantioselectivity with up to 24% e.e. for acid (**5**).

**Scheme 3:**
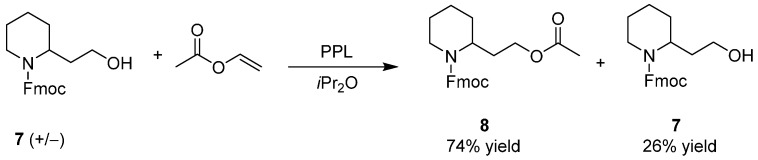
Enzymatic kinetic resolutions of racemic **7**. PPL: porcine pancreas lipase. Fmoc: 9-fluorenylmethyl carbamate.

In 1994, a first example of exploitation of the porcine pancreas lipase (PPL) for the resolution of 9-fluorenylmethyl carbamate (*N*-Fmoc) 2-piperidineethanol (**7**) was reported in a Japanese patent [33]: vinyl acetate was used as the acylating agent and diisopropyl ether as solvent (Scheme 3) (in the English abstract are not indicated the e.e. of both the enantioenriched **8** and **7**).

**Scheme 4:**
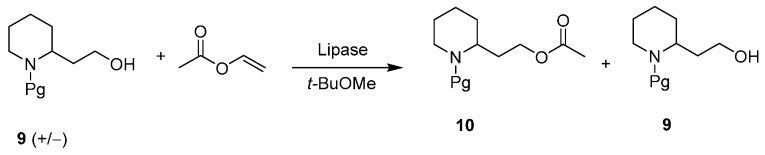
Enzymatic enantioselective acylation of racemic **9**. Pg: protecting group.

Finally, in 2003, a comprehensive study on the enantioselective acylation of the residual primary alcohol (Scheme 4) by lipases was successfully realized [34]. A first screening of over 20 different lipase preparations was performed at 45 °C; an organic solvent (methyl *tert*-butyl ether) was selected as solvent as it is well-known that the enzymatic performances (activity and selectivity) can be enhanced greatly using organic solvents as a reaction medium, rather than their natural aqueous environments [35]; the protective groups of amino group tested were Boc, Cbz, and Fmoc.

This preliminary screening underlined that reaction enantioselectivities were generally quite low. The crude porcine pancreatic preparation possessed an opposite enantioselectivity compared to all the other lipases and Lipase PS was the more selective enzyme among the “(*R*)-lipases”. Comparing the three protective groups, *N*-Boc derivative was a slightly better substrate. A new screening was directed to pointing out the effect of different solvents for the reaction of *N*-Boc derivative catalyzed by Lipase PS and PPL: hexane was a slightly better solvent for the former while methyl *tert*-butyl ether remained the solvent of choice for the latter.

**Scheme 5:**
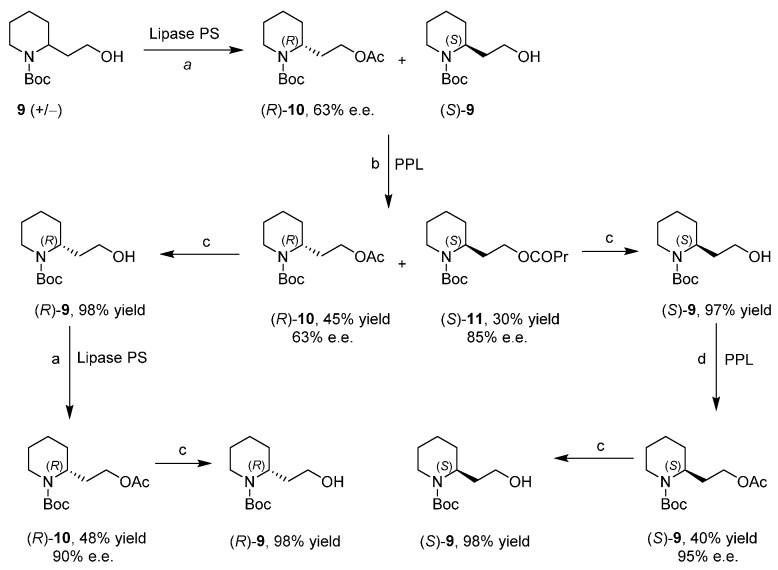
Optimized protocol. (**a**) AcOCH=CH_2_, hexane, 20 °C, 190 min (45%); (**b**) PrCOOCH=CH_2_, MTBE, 20 °C, 11.5 h (30%), flash chromatography (AcOEt-hexane 1:9); (**c**) CH_3_OH, Na_2_CO_3_; and (**d**) AcOCH=CH_2_, MTBE, 20 °C, 46 h (48%). PPL: porcine pancreas lipase; PS: Lipase PS; Pr: propyl; Ac: acetyl; Boc: *tert*-butyl carbamate.

Finally, an optimized protocol was designed based on the observed complementary enantioselectivity of the two enzymes (Scheme 5). The racemic mixture **9** was acetylated under the best condition for Lipase PS; after that enzyme is filtered and reagent removed, the crude mixture was butanoylated by the PPL under the best condition for this enzyme. In this way, the low selectivity of the two lipases was enhanced. The final mixture, composed by enantioenriched acetate (*R*)-**10**, butanoate (*S*)-**11** and the unreacted almost racemic **9**, was easily purified by flash chromatography. Hydrolysis of the two esters was followed by a second step of acetylation on the two enantioenriched alcohols by the two lipases; after hydrolysis, both alcohols with high e.e. were obtained.

## 3. An Overview of the Synthetic Strategies in the Synthesis of Alkaloids Starting from Either Enantiopure 1 or 2

The development of suitable methods for accessing both enantiomers of **1** opened the path to the total synthesis of a wide range of natural compounds with high interest in medicinal chemistry; in an early stage, some of these syntheses were performed starting with racemic **1**.

**Scheme 6:**
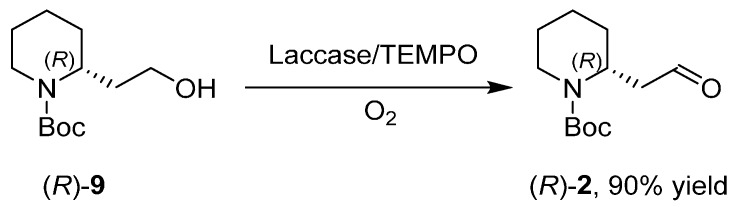
Oxidation reaction mediated by laccase. TEMPO: 2,2,6,6-tetramethyl-1-piperidinyloxy.

As previously mentioned, **2** is the main derivative of 2-piperidineethanol used in total synthesis where the stereogenic center at the C-2 position would readily encode the remaining stereochemical information into the final target. It is usually synthesized by Swern oxidation, but some troubles affect the recovery of the product and the scale up of the reaction. An enzymatic approach at the oxidation reaction of (*R*)-**9** was realized by exploiting laccases [36], either from *Trametes pubescens* or from *Myceliophtora termophyla*, TEMPO (2,2,6,6-tetramethyl-1-piperidinyloxy) as a mediator, and molecular oxygen of the air as the primary oxidant (Scheme 6).

### 3.1. Enantioselective Synthesis of the Alkaloid (+)-Aloperine

**Scheme 7:**
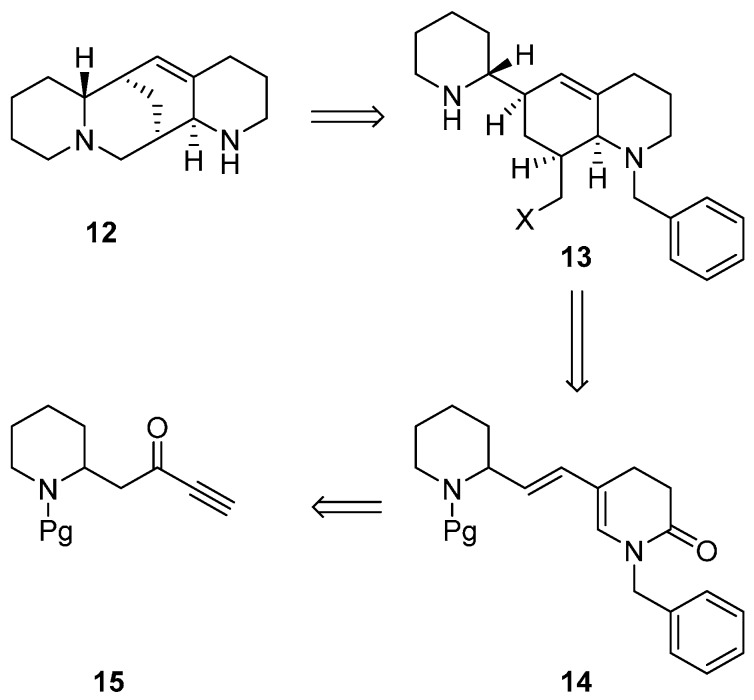
Retrosynthetic analysis of the synthesis of aloperine.

Aloperine (**12**) (Scheme 7) is a member of a small family of C_15_ alkaloids that is extracted from the seeds and leaves of *Sophora alopecuroides*, a shrub that grows in China and Russia, and later from *Leptorhabdos parviflor Benth.* Its pharmacological activity spreads between cardiovascular, anti-inflammatory, and anti-allergic features producing an intense synthetic interest in this molecule. In 2002, a synthesis of racemic **12** was realized [37]; the synthetic strategy (Scheme 7) relied on an efficient intermolecular Diels-Alder reaction as crucial step where derivative **15** was a key building block for the synthesis of the *N*-acylaminodiene **14**.

**Scheme 8:**
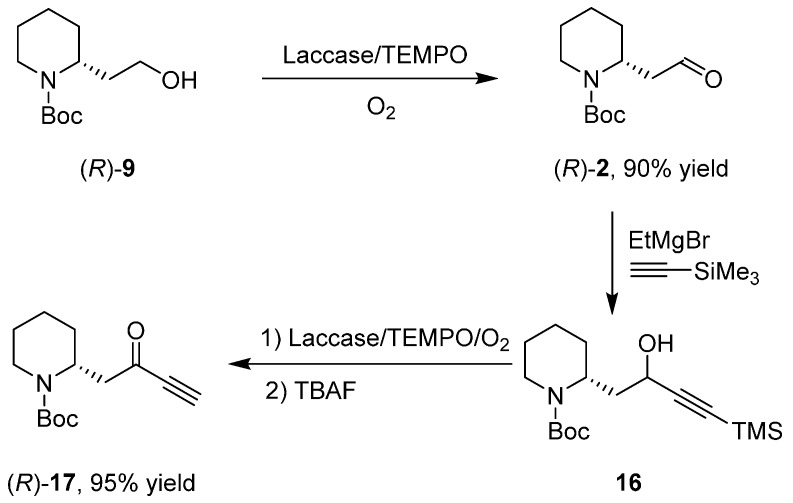
Synthesis of intermediate (*R*)-17. TBAF: tetrabutylammonium fluoride. TMS: trimethylsilyl.

In 2004, the corresponding enantioselective variant [36] was performed starting from (*R*)-**9** for the synthesis of (*R*)-**17** (Scheme 8); two consecutive enzymatic oxidations mediated by laccase were set for both the synthesis of aldehyde (*R*)-**2** and ketone (*R*)-**17**.

### 3.2. Enantioselective Synthesis of the Alkaloid Boehmeriasin A

**Scheme 9:**
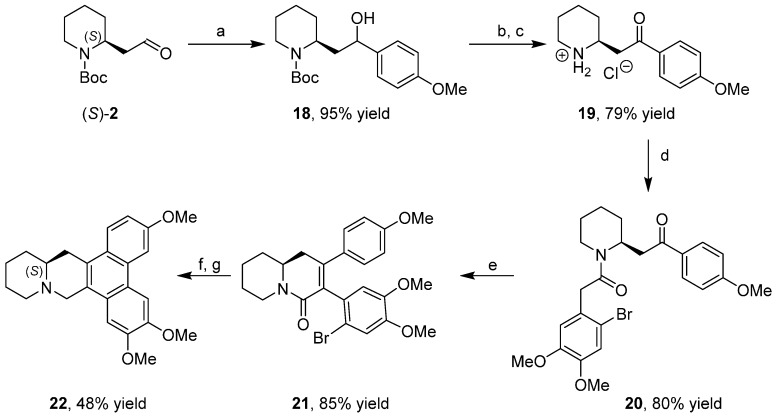
Synthesis of the alkaloid boehmeriasin A. (**a**) (4-methoxyphenyl)magnesium bromide; (**b**) DMP; (**c**) TMSCl, MeOH; (**d**) 2-bromo-4,5-dimethoxyphenylacetic acid, DIPEA, 1-(Bis(dimethylamino) methylene)-1H-1,2,3-triazolo(4,5-b)pyridinium 3-oxid hexafluorophosphate (HATU); (**e**) KOH, EtOH; (**f**) Pd(OAc)_2_, K_2_CO_3_, 2′-(diphenylphosphino)-*N*,*N*′-dimethyl-(1,1′-biphenyl)-2-amine; and (**g**) LiAlH_4_.

Boehmeriasin A (**22**) is a phenanthroquinolizidine alkaloid extracted from *Boehmeria siamensis* *Craib*. It possesses a strong cytotoxic activity, more potent than taxol, against 12 cell lines from six panels of cancer including breast, kidney, prostate, colon, lung cancer, and leukemia, with topoisomerases and SIRT2 as involved targets. Recently, the synthesis of enantiopure **22** [38] was accomplished starting from aldehyde (*S*)-**2** (Scheme 9).

The synthetic sequence begins with the Grignard addition to the enantiopure aldehyde (*S*)-**2** followed by oxidation with DMP (Dess-Martin periodinane), deprotection of the Boc group, *N*-acylation of the amine, intramolecular aldol-type condensation, and palladium-catalyzed intramolecular coupling and reduction of the amide.

Recently, a quite similar synthetic strategy [39] was used for the synthesis of sulfonamide analogues of **22** as potent and orally active antitumor agents.

### 3.3. Enantioselective Synthesis of the Alkaloid Dumetorine

**Scheme 10:**
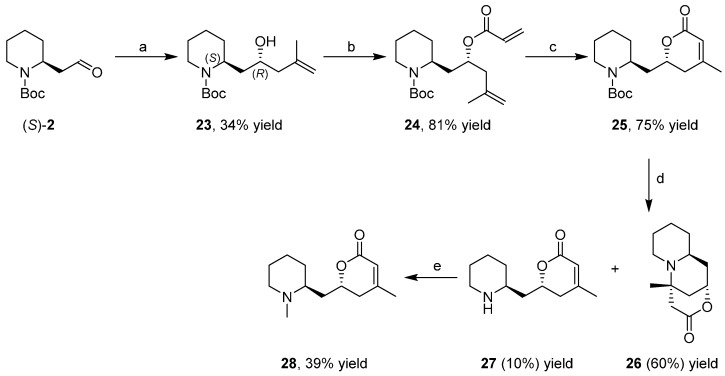
Enantioselective synthesis of the alkaloid dumetorine. (**a**) CH_2_=CHCH_3_CH_2_MgBr, (+)-β-methoxydiisopinocamphenylborane; (**b**) CH_2_=CHCOCl; (**c**) Grubb’s II catalyst; (**d**) TFA (trifluoroacetic acid); and (**e**) CH_2_O, NaBH_3_CN.

Dumetorine (**28**) is an alkaloid with piperidine structural unit that is extracted from the tubers of *Dioscorea dumetorum Pax* and used in folk medicine and arrow poisons. The enantioselective synthesis [40] was established on few key synthetic steps (Scheme 10) starting from the enantiopure aldehyde (*S*)-**2**. The second stereocenter was achieved by an asymmetric allylboration reaction with β-allyl-diisopinocamphenylborane, obtaining exclusively the required compound **23** (diastereoisomeric excess (de) > 95%). After acylation with acryloyl chloride, the dihydropyran ring was synthesized by ring-closing metathesis reaction with the second generation Grubbs catalyst. Unfortunately, the removal of the Boc group with TFA (trifluoroacetic acid) involved the formation of the byproduct **26**, as the main component of the reaction mixture, and the desired free amine **27** in small amounts (10%). Finally, *N*-methylation was carried out by reductive amination with CH_2_O in the presence of NaBH_3_CN to give the target natural (+)-**28**.

Recently, for the improvement of the synthesis, a sequence of five separate synthetic steps, was performed by flow chemistry technology with the advantages of minimal workup and purification [41]. Packed columns containing appropriate immobilized reagents or scavenger materials were used. The starting compound was the enantiopure alcohol (*S*)-**9** that was converted into the corresponding aldehyde (*S*)-**2** by continuously cycling the flow stream through a polymer-supported 2-iodoxybenzamide pre-packed column. In the second step, the insertion of the allyl group was realized by reaction with the Grignard reagent 2-methyl-allylmagnesium bromide; the two diastereoisomers, formed almost in 1:1 ratio, were purified on a silica gel cartridge to give the desired diastereoisomer **23**. After acylation with acryloyl chloride, the ring-closing metathesis reaction was realized by a new PEG (polyethylene glycol)-supported Hoveyda catalyst working in homogeneous conditions ensured by the solubility of the catalyst in CH_2_Cl_2_, maintaining the possibility of simple catalyst recovery by solvent-promoted precipitation and filtration. The inherent instability of amine **27** was the main drawback of the early synthetic sequence (Scheme 10) since Michael addition of the secondary amine to the α,β-unsaturated lactone ring caused the formation of the byproduct **26** and significantly reduced the yield of the final reductive amination (39%). A definitive breakthrough was established by converting directly Boc protected **25** in final product **28** through an Eschweiler–Clarke reaction with formaldehyde in the presence of formic acid at high temperature: flash heating with a rapid warming to 140 °C gave a clean product with a yield of 92%.

### 3.4. Enantioselective Synthesis of the Alkaloids Sedamine, Allosedamine, Sedridines and Ethylnorlobelols

**Scheme 11:**
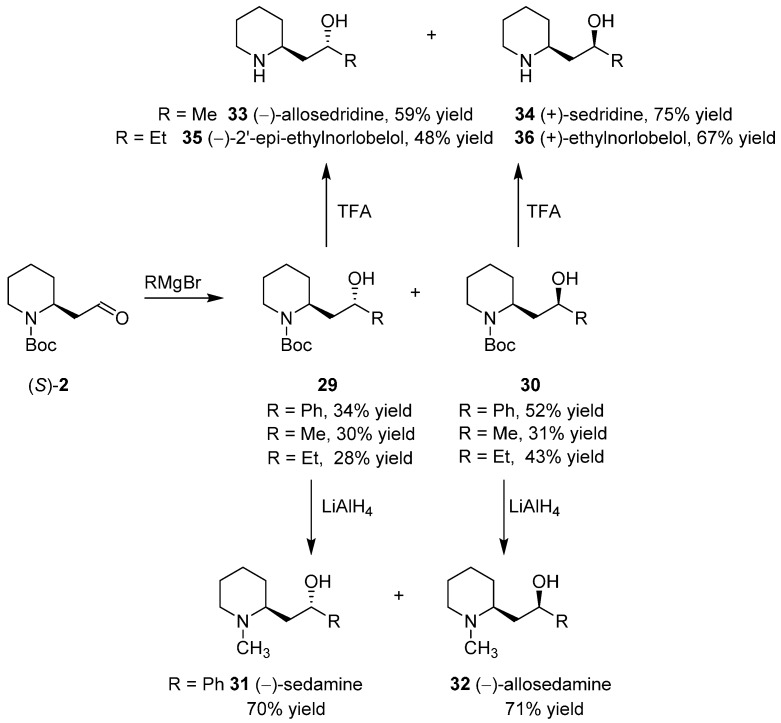
Enantioselective synthesis of the alkaloids sedamine, allosedamine, sedridines, and ethylnorlobelols.

The structure of several alkaloids are easily recognized as derivatives of the reaction of **2** with an appropriate Grignard reagent. Indeed, starting from the enantiopure (*S*)-**2**, two diastereoisomeric alcohols **29** and **30** were generated (Scheme 11). Their Boc groups were easily converted into methyl groups by reduction with LiAlH_4_ [34] affording, for R = Ph, respectively, (−)-sedamine (**31**) and (−)-allosedamine (**32**); alternatively, **31** was synthesized from **29**, through flow chemistry technology, by the protocol developed for the previously outlined Eschweiler–Clarke reaction [41].

On the other hand, removing the Boc group from **29** and **30** afforded respectively (−)-allosedridine (**33**) and (+)-sedridine (**34**), for R = Me, (−)-2′-epi-ethylnorlobelol (**35**) and (+)-ethylnorlobelol (**36**), for R = Et [42].

### 3.5. Synthesis of the Enantiopure Alkaloids Coniine and Epidihydropinidine

**Scheme 12:**
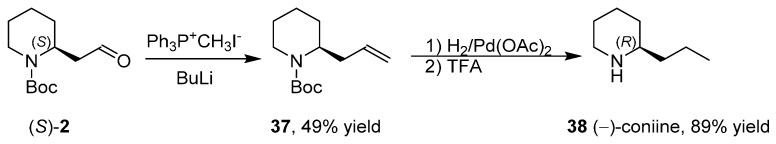
Synthesis of the alkaloid (−)-coniine.

Coniine (**38**) is the major alkaloid extracted from *Conium maculatum* (poison hemlock) and responsible for its toxicity. It was synthesized from the enantiopure (*S*)-**2** (Scheme 12) by elongating the chain at the 2-position of the piperidine ring through a Wittig reaction, hydrogenation of the double bound and removal of the Boc group [42].

**Scheme 13:**
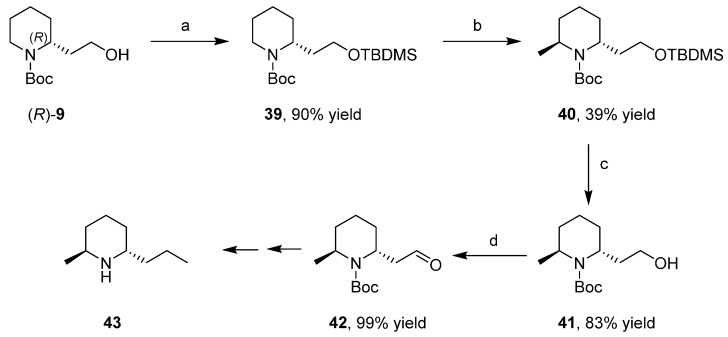
Enantioselective synthesis of the alkaloid (−)-epidihydropinidine. (**a**) imidazole, *tert*-butyldimethylsilyl chloride (TBDMSCl); (**b**) *N*,*N*,*N*′,*N*′-Tetramethylethylenediamine (TMEDA), *sec*-BuLi, Et_2_O, −78 °C, MeSO_4_; (**c**) Na_2_CO_3_, MeOH; and (**d**) Dess-Martin reagent.

Closely related to coniine is the alkaloid epidihydropinidine (**43**) that shared the same synthetic steps for the building of the propyl group [40]. The key synthetic step was the Beak’s *N*-Boc-piperidine α-lithiation/alkylation methodology for the introduction of the methyl group at the C-6 position; TBDMS protection of the hydroxyl group of (*R*)-**9** was important in order to keep the C-2 chain at a distance from the reactive center (Scheme 13). After deprotection and Dess–Martin oxidation to aldehyde **42**, the synthesis proceeded alike Scheme 12.

### 3.6. Enantioselective Synthesis of Polycyclic Piperidine Derivatives

**Scheme 14:**
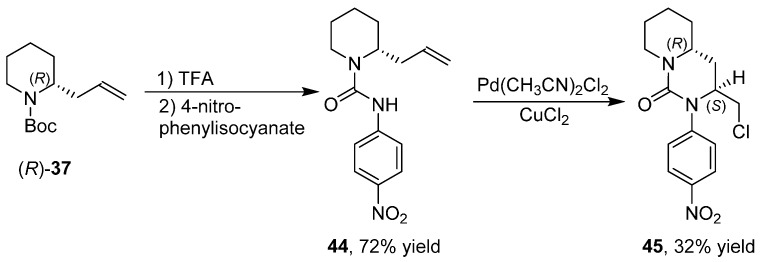
Enantioselective synthesis of bicyclic derivative **45**.

The changeover of the aldehyde group of **2** into the carbon–carbon double bond of **37** allowed a switch of reactivity by exploiting reactions affecting the alkene group [43] such as intramolecular Heck reactions, 1,3-dipolar cycloadditions, ring closing metathesis and intramolecular chloroamination reactions, reaching an increased molecular complexity. Indeed, bicyclic derivative **45** was obtained by reaction of urea **44** using Pd(CH_3_CN)_2_Cl_2_ as a catalyst and CuCl_2_ as an oxidant (Scheme 14).

**Scheme 15:**
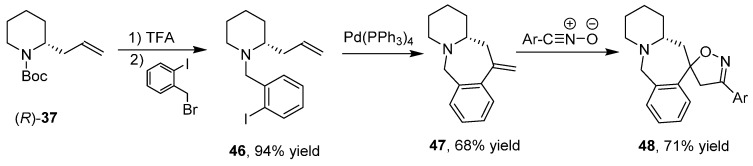
Synthesis of derivative **48**.

Combining more reactions together in order to modifying the carbon–carbon double bond before the 1,3-dipolar cycloaddition, allowed the stereoselective synthesis of fused tricyclic derivatives with up to three stereogenic centers. Then, aryl derivative **46** was subjected to an intramolecular Heck reaction (Scheme 15) obtaining the bicyclic alkene **47** that, after 1,3-dipolar cycloaddition, gave the tricyclic spiro derivative **48** as mixture of two diastereoisomers.

**Scheme 16:**
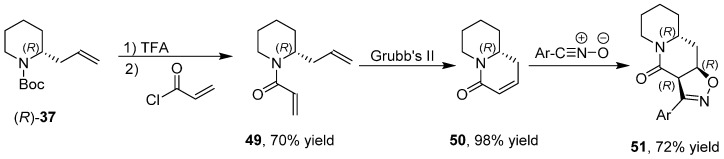
Synthesis of derivative **51**.

Likewise, derivative **49** was subjected to ring-closing metathesis (Scheme 16) by a Grubbs catalyst of second generation obtaining the bicyclic unsaturated amide **50** that after 1,3-dipolar cycloaddition gave the tricyclic derivative **51** as single diastereoisomer.

## 4. A General Overview of the Structures of Alkaloids Synthesized from both 1 or 2

Further examples of natural compounds synthesized starting from both enantiopure **1** or **2** are shortly exemplified in this subsection; the substructure derived from **1** is pointed out in red.

### 4.1. Enantioselective Synthesis of the Alkaloids (−)-Oncinotine, (−)-Isooncinotine, and (−)-Stellettamide B

**Figure 2:**
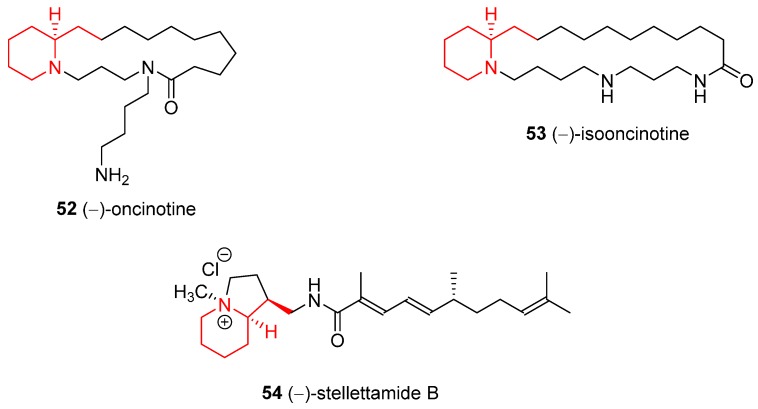
Structures of the alkaloids (−)-oncinotine, (−)-isooncinotine, and (−)-stellettamide B.

(−)-Oncinotine (**52**) and (−)-isooncinotine (**53**) (Figure 2) are a group of isomeric polyamine alkaloids which are characterized by macrocyclic lactams containing the biogenetic base spermidine; they are isolated from the stem bark of *Oncinotis nitida* (Apocynaceae). **52** was synthesized in 1996 [19] starting from *N*-Cbz **2** instead. **53** was synthesized in 2007 [44] from **1**. Stellettamide B (**54**) (Figure 2) is extracted from a marine sponge of the genus *Stelletta* and showed antifungal and cytotoxic activity; it was synthesized in 2001 [20] from **1**.

### 4.2. Enantioselective Synthesis of the Alkaloids (+)-Vertine and (+)-Lythrine

**Figure 3:**
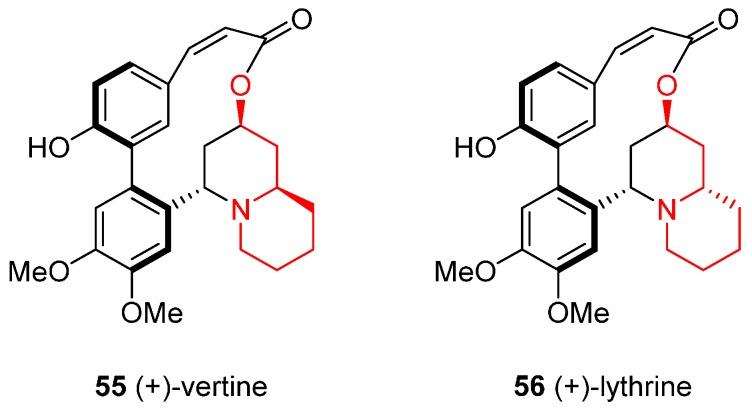
Structures of the alkaloids (+)-vertine and (+)-lythrine.

The azabicyclic skeleton of quinolizidine is a relevant structural subunit present in numerous alkaloids and can be built starting from **1**. (+)-Vertine (**55**) and (+)-lythrine (**56**) are examples of this class of natural compounds (Figure 3); they are extracted from *Decodon verticillatus* and displayed a wide range of biological activities, such as anti-inflammatory, sedative, and antispasmodic actions; recently, was realized their total synthesis starting from **1** [45].

### 4.3. Enantioselective Synthesis of the Alkaloids (+)-Myrtine, (−)-Lupinine, and (+)-Epiepiquinamide

**Figure 4:**
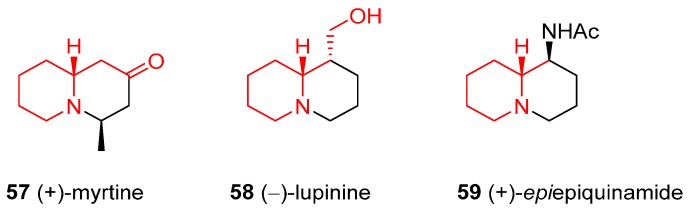
Structures of the alkaloids (+)-myrtine, (−)-lupinine, and (+)-*epi*epiquinamide.

(+)-Myrtine (**57**), isolated from *Vaccinium myrtillus*, (−)-lupinine (**58**), isolated from the yellow lupin seeds (*Lupinus luteus*), as well as (+)-epiepiquinamide (**59**), isolated in 2003 from the poison frog *Epipedobates tricolor*, share a quite comparable structure, although they originate from different natural sources (Figure 4). They were synthesized in 2011 from *N*-Boc **2** [46].

### 4.4. Enantioselective Synthesis of the Alkaloids (−)-Cermizine C, (−)-Senepodine G and (+)-Cermizine D

**Figure 5:**
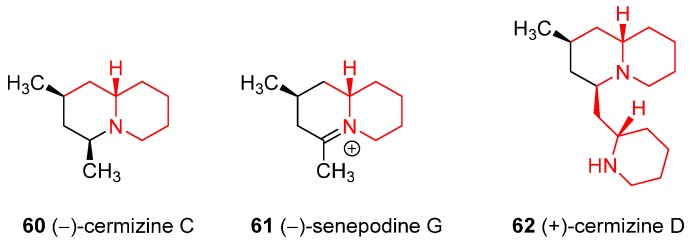
Structures of the alkaloids (−)-cermizine C, (−)-senepodine G, and (+)-cermizine D.

From the family of lycopodium alkaloids are worth to mention (−)-Cermizine C (**60**) and (+)-cermizine D (**62**), both isolated from the club moss *Lycopodium cernuum* and the related alkaloid (−)-senepodine G (**61**) from the club moss *Lycopodium chinense* (Figure 5); the second latter displaced modest cytotoxicity against murin lymphoma L1210 cells. **60** was synthesized from **61** by reduction with NaBH_4_, while the synthesis of the latter was accomplished starting from **1** [47,48]. Enantioselective synthesis of **62** exploited the use of *N*-Boc **2** as common intermediate to access two of the three piperidine rings [49].

### 4.5. Enantioselective Synthesis of the Alkaloids Psylloborine A and Isopsylloborine A

**Figure 6:**
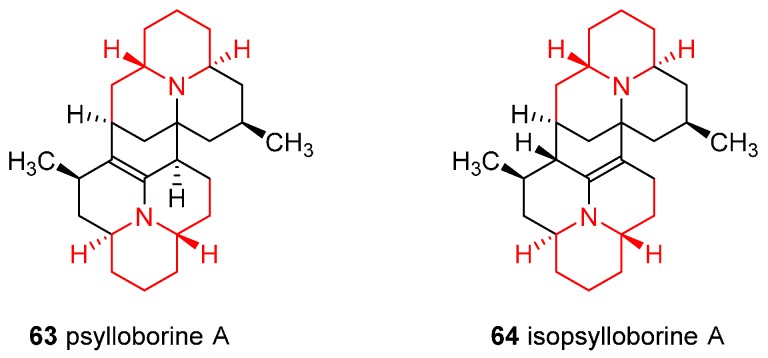
Structures of the alkaloids psylloborine A and isopsylloborine A.

The complex dimers psylloborine A (**63**) and isopsylloborine A (**64**) (Figure 6) belong to coccinellid family of alkaloids, materials secreted by numerous species of ladybugs as defensive compounds when provoked. In the synthesis, *N*-Boc **9** was identified as a common starting point for the two tricyclic intermediate of the dimerization reaction [50]; moreover, the synthetic strategy allowed the total syntheses of eight monomeric members of these class of alkaloids.

### 4.6. Enantioselective Synthesis of the Alkaloids Tetraponerines T3, T4, T7, and T8

**Figure 7:**
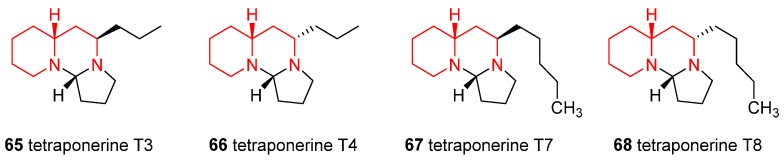
Structures of the tetraponerines T3, T4, T7, and T8.

Tetraponerines are a family of alkaloids that Pseudomyrmecine ants of the genus *Tetraponera* segregate against their enemies; their structures possess a singular aminal moiety inside a fused tricycle. They act as paralyzing venoms since are efficient inhibitors of a range of nAChRs (nicotinic acetylcholine receptors). Recently, enantioselective synthesis of the tetraponerines T3 (**65**), T4 (**66**), T7 (**67**), and T8 (**68**) (Figure 7) was achieved starting from *N*-Cbz **2** [51,52]. Their anticancer activity, against four different carcinoma human cell lines was examined showing a promising cytotoxic activity of **67** against breast carcinoma cell line MCF-7.

### 4.7. Enantioselective Synthesis of the Alkaloids (+)-Conhydrine and 4-(2-(Piperidin-2-yl)ethoxy)quinoline Derivatives

**Figure 8:**
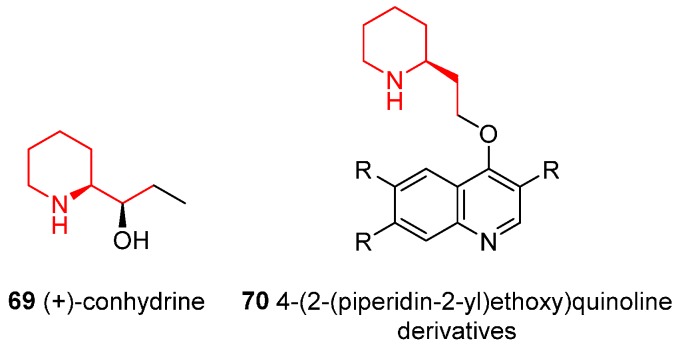
Structure of (+)-conhydrine and general structure of 4-(2-(piperidin-2-yl)ethoxy)quinoline derivatives.

Other alkaloids that contain the piperidine unit are: (+)-conhydrine (**69**) (Figure 8) [53], which was isolated in 1856 from the seeds and leaves of the poisonous plant *Conium maculatum* L. and is effectively in the treatment of cognitive disorders since it has been shown to display memory-enhancing properties; and 4-(2-(piperidin-2-yl)ethoxy)quinoline derivatives (**70**), as selective agonists of somatostatin receptor subtype 2 (Figure 8) [54].

## 5. Conclusions

The discovery of both synthetic and enzymatic methods for the obtaining of enantiopure **1** opens the way for the use of this compound as a vital starting material for the enantioselective synthesis of natural and synthetic compounds. This review highlights the synthesis of natural alkaloids starting from both enantiopure 2-piperidineethanol and its corresponding derivative N-protected aldehyde **2**.
